# Antipsychotic drug exposure and risk of pulmonary embolism: a population-based, nested case–control study

**DOI:** 10.1186/s12888-015-0479-9

**Published:** 2015-04-29

**Authors:** Valentino Conti, Mauro Venegoni, Alfredo Cocci, Ida Fortino, Antonio Lora, Corrado Barbui

**Affiliations:** Regional Centre for Pharmacovigilance, Lombardy Region, Milano, Italy; Unit of Community Health Services, Regional Health Ministry, Lombardy Region, Milan, Italy; Department of Mental Health, Lecco, Italy; WHO Collaborating Centre for Research and Training in Mental Health and Service Evaluation, Department of Public Health and Community Medicine, Section of Psychiatry, University of Verona, Verona, Italy

## Abstract

**Background:**

Only three observational studies investigated whether exposure to antipsychotics is associated with an increased risk of pulmonary embolism, with conflicting results. This study was therefore carried out to establish the risk of pulmonary embolism associated with antipsychotic drugs, and to ascertain the risk associated with first- and second-generation antipsychotic drugs, and with exposure to individual drugs.

**Methods:**

We identified 84,253 adult individuals who began antipsychotic treatment in a large Italian health care system. Cases were all cohort members who were hospitalized for non-fatal or fatal pulmonary embolism during follow-up. Up to 20 controls for each case were extracted from the study cohort using incidence density sampling and matched by age at cohort entry and gender. Each individual was classified as current, recent or past antipsychotic user. The occurrence non-fatal or fatal pulmonary embolism was the outcome of interest.

**Results:**

Compared to past use, current antipsychotic use more than double the risk of pulmonary embolism (odds ratio 2.31, 95% confidence interval 1.16 to 4.59), while recent use did not increase the risk. Both conventional and atypical antipsychotic exposure was associated with an increase in risk, and the concomitant use of both classes increased the risk of four times (odds ratio 4.21, 95% confidence interval 1.53 to 11.59).

**Conclusions:**

Adding the results of this case–control study to a recent meta-analysis of three observational studies substantially changed the overall estimate, which now indicates that antipsychotic exposure significantly increases the risk of pulmonary embolism.

**Electronic supplementary material:**

The online version of this article (doi:10.1186/s12888-015-0479-9) contains supplementary material, which is available to authorized users.

## Background

Pulmonary embolism (PE) is a significant cause of morbidity and mortality, occurring at an estimated 95 cases per 100,000 patient-years [1,2]. The severity of PE ranges from asymptomatic, incidentally discovered subsegmental thrombi to hemodynamically unstable, life-threatening episodes, and sudden death [3,4].

In 1997 Walker and colleagues conducted an epidemiological study suggesting that exposure to clozapine, an agent belonging to the group of the so-called second-generation antipsychotic (AP) drugs, significantly increased the risk of PE mortality [5]. Since then, other epidemiological cohort and case control studies provided additional data on this association [6]. A recent systematic review and meta-analysis identified 13 studies, of which only three investigated PE outcomes, while ten estimated the association between AP exposure and risk of a composite outcome which included deep venous thrombosis, femoral vein thrombi, popliteal vein thrombi, iliac vein thrombi, deep vessels of lower extremity thrombophlebitis, and pulmonary embolism [7]. Analysis of the three PE studies failed to detect a significant association between exposure to APs and risk of PE outcomes (odds ratio 4.90, 95% confidence interval 0.77 to 30.98), but the confidence interval was very wide and included the possibility of substantial harm [7].

Therefore, we investigated whether exposure to AP drugs is associated with an increased risk of PE, and ascertained the risk associated with first- and second-generation AP drugs, and with exposure to individual drugs. We carried out a nested case–control study using a large administrative database in the health system of Lombardy, a region located in northern Italy. As secondary aim, we updated the published meta-analysis with the results of this study, using the same methodology.

## Methods

### Setting and data source

Lombardy is the largest and the most affluent region in Italy, with a population of around 10 million inhabitants. Lombardy is located in the northernmost part of the country and includes the metropolitan area of Milan, Italy’s second largest city.

The data used for this study were retrieved from the Regional Health Service (RHS) databases of Lombardy [8,9]. These databases include: i) demographic and administrative information on all residents in the Lombardy region; ii) all community (outside the hospital) drug prescriptions reimbursed by the RHS; iii) all public and private hospital discharge forms, with diagnoses according to the ICD-9-CM. According to local regulations on administrative database analyses, no formal approval of the study protocol was required; however, as previously reported for similar analyses [8], to preserve patient privacy, the identification codes from all the databases were converted to anonymous codes, and the conversion table was then destroyed.

### Study population and design

From the regional prescription database we identified all individuals aged ≥ 18 starting a new treatment with AP drugs from 1 January 2012 to 31 December 2013. AP drugs were defined as all drugs belonging to the N05A Anatomical Therapeutic Chemical Classification (ATC) group (with the exception of N05AN, lithium). New AP users were individuals with no AP prescriptions in the 12 months before the first prescription issued in the study period. From the group of new AP users, patients with a recorded diagnosis from hospital discharge forms of neoplasm (ICD9-CM 140–239), hip fracture (81.xx), pulmonary embolism or deep vein thrombosis (415.xx, 453.xx, 451.1x) in the year before the first AP prescription were excluded.

Each member of the cohort was followed from the first AP prescription until the earliest of the following event: outcome of interest (PE), death for any cause, emigration and end of follow-up.

### Selection of cases and controls

Cases were all cohort members who were hospitalized for non-fatal or fatal PE during follow-up (ICD9-CM codes 415.xx). For cases with more than one hospital admission for PE during follow-up, we selected the first record of PE. Up to 20 controls for each case were extracted from the study cohort using incidence density sampling and matched by age at cohort entry and gender. All controls were alive and at risk of the outcome of interest at the date of the first PE in their matched case (index date). Cases and controls with pregnancy (ICD9-CM codes 630–677), leg/hip fracture or a diagnosis of neoplasm in the three months before the index date were excluded. In agreement with Parker and colleagues [10], controls with any prescriptions of warfarin or other antithrombotic drug use (ATC code B01AA03/B01AA07) before the index date were excluded. For cases, prescriptions for warfarin or other antithrombotic drugs in the six weeks before the index date did not trigger exclusion as they could plausibly be treatment for the index event itself [10]; however we excluded cases with any use of warfarin or other antithrombotic drugs earlier than this six week period.

### Assessment of AP exposure

We assessed exposure to AP drugs on the basis of prescriptions on or before the index date. Each individual was classified for exposure to AP as current user (one or more prescriptions within three months before index date); recent user (one or more prescriptions in the period between 4 and 12 months before index date); past user (one or more prescriptions 13 months or more before index date). Exposure during the past three months was classified according to AP type (conventional only, atypical only, both), formulation (oral, acute injection, long-acting injection), number of AP scripts, number of different AP prescribed, and overall amount of AP exposure, calculated by dividing the total amount of prescribed drug by the recommended daily dose, according to each agent’s defined daily dose (DDD). The DDD is a theoretical unit of measurement defined as the assumed average maintenance daily dose for a drug, used for its main indication in adults [11].

### Data analysis

Using the whole study cohort, we estimated the rate ratios with 95% confidence intervals (CIs) for the association between PE and AP exposure, taking past use as the reference category.

For the case–control analysis, cases and controls were compared using chi-square test, for categorical data, and t test for continuous data. We undertook multiple conditional logistic regression to estimate the odds ratio (OR), with 95% CIs, for the association between PE and AP use. Subgroup analyses were performed for age (≥65 years, < 65 years) and gender. ORs were adjusted for the following confounding variables: psychiatric hospitalization (ICD9-CM code 290–319); hospitalization for coronary heart disease (410.xx-414.xx), heart failure (428.xx), stroke (431.xx, 433.xx-436.xx), chronic liver disease (570–573), chronic renal disease (584–586), Parkinson’s disease (ICD9-CM code 332 or drug prescription with ATC code N04), diabetes (ICD9-CM code 249–250 or drug prescription with ATC code A10); prescription of lithium (ATC code N05AN01), statins (C10AA), NSAIDs (M01A), antiplatelet treatment (A01AD05, B01AC06, N02BA01, B01AB, B01AC04, B01AC05), and antihypertensive agents (C02, C03,C07,C08,C09). All covariates were considered in the 12 months before index date. We did not adjust for varicose veins, inflammatory bowel disease, and use of hormone replacement therapy, as suggested by Parker [10], as only very few cases with these disorders were recorded.

Numbers needed to harm and extra cases per 10 000 treated were estimated, using the incidence of PE in the past users and the adjusted ORs from current users.

Finally, in order to provide a summary of the whole evidence base, we added the results of the present case–control study to a recently published meta-analysis of observational studies on the risk of PE outcomes associated with AP exposure (Additional file 1) [7]. A meta-analysis influence test that eliminated each of the included studies one at a time was carried out to test for possibly excessive influence of individual studies.

Data management and analyses were carried out using SAS statistical package (SAS Institute, Cary, NC), version 9.2 and Comprehensive Meta-Analysis version 2: the PHREG procedure with STRATA statement was used to fit the conditional logistic regression models. All tests were two-tailed, and results were considered significant at p < 0.05.

## Results

### Study cohort

**Figure 1:**
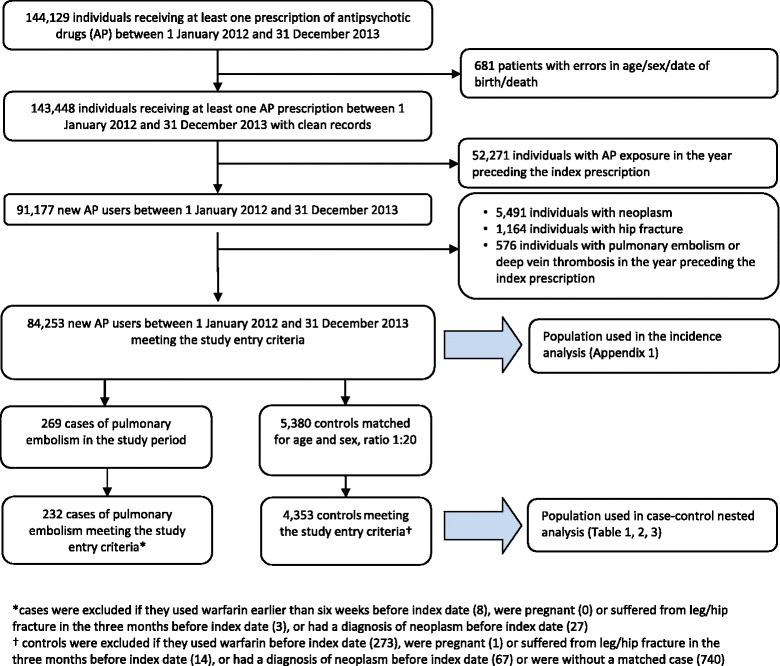
Flow chart of inclusion and exclusion criteria of cohort of new treated with antipsychotic drugs in Lombardy Region from 2012 to 2013.

During the study period 144,129 individuals received one or more AP prescriptions; of these, 84,253 were new users and fulfilled the inclusion criteria (Figure 1). The study cohort accumulated 88,184 person-years of follow-up (a mean of 1 year per patient) and generated 269 PE events, with an incident rate of 305 cases for 100 000 person-years at risk (Additional file 2).

### Cases and controls

Of the 269 individuals with a recorded PE event, 232 were eligible as cases, and were matched with 4,353 controls from the same study cohort (Figure 1). Cases and controls were well matched by age and gender (Table 1). Cases were more likely than controls to have admissions for psychiatric disorders and for other medical conditions, and were more often prescribed antiplatelet and antihypertensive treatment.

**Table 1:** **Characteristics of cases (patients with first record of pulmonary embolism) and matched controls at index date**

	**Cases (n = 232)**	**Controls (n = 4353)**	P value*
Mean age ± SD	77.1 ± 13.5	76.9 ± 13.7	0.798
Age ≥ 65	194 (83.6%)	3,610 (82.9%)	
Age < 65	38 (16.4%)	743 (17.1%)	
Women	157 (67.7%)	2,950 (67.8%)	0.975
Men	75 (32.3%)	1,403 (32.2%)	
Hospitalization§			
Mental disorders	72 (31.0%)	468 (10.8%)	<.001
Coronary heart disease	22 (9.5%)	114 (2.6%)	<.001
Heart failure	21 (9.1%)	118 (2.7%)	<.001
Stroke	12 (5.2%)	108 (2.5%)	0.012
Chronic liver disease	5 (2.2%)	19 (0.4%)	0.000
Parkinson’s disease	32 (13.8%)	481 (11.0%)	0.197
Chronic renal disease	20 (8.6%)	90 (2.1%)	<.001
Drug use§			
Lithium	5 (2.2%)	84 (1.9%)	0.808
Statins	39 (16.8%)	744 (17.1%)	0.912
NSAIDs	67 (28.9%)	1,036 (23.8%)	0.078
Antiplatelet treatment	123 (53.0%)	1,981 (45.5%)	0.025
Anti-diabetic	34 (14.7%)	613 (14.1%)	0.807
Antihypertensive agents	155 (66.8%)	2,608 (59.9%)	0.036

*chi-square statistics.

§ in the 12 months preceding index date.

NSAIDs **=** non-steroidal anti-inflammatory drugs.

### Risk associated with AP drugs

Compared to past use, current AP use more than double the risk of PE (OR 2.31, 95% CI 1.16 to 4.59), while recent use did not increase with the risk of PE outcomes (OR 0.96, 95% CI 0.44 to 2.07) (Table 2). Both conventional and atypical AP exposure was associated with an increase in risk, and the concomitant use of both AP classes increased the risk of four times (OR 4.21, 95% CI 1.53 to 11.59) (Table 2). The total number of AP prescribed, and the overall amount of AP prescribed, expressed as multiples of the DDD, were also significant risk factors of PE (Table 2).

**Table 2:** **Risk of pulmonary embolism associated with antipsychotic drug use**

	**Cases (n = 232)**	**Controls (n** = **4353)**	**Unadjusted odds ratio (95% CI)**	**P value**	Adjusted* odds ratio (95% CI)	**P value**
Category of antipsychotic exposure:						
Current	195 (84.1%)	2,956 (67.9%)	2.91 (1.48 - 5.73)	0.002	2.31 (1.16 - 4.59)	0.017
Recent	28 (12.1%)	1,002 (23.0%)	1.24 (0.58 - 2.64)	0.585	0.96 (0.44 - 2.07)	0.910
Past^∫^	9 (3.9%)	395 (9.1%)	1		1	
Class of antipsychotic received: (within past 3 months)						
Conventional only	53 (22.8%)	507 (11.6%)	4.67 (2.27 - 9.61)	<0.001	3.52 (1.69 - 7.35)	0.001
Atypical only	134 (57.8%)	2,386 (54.8%)	2.49 (1.26 - 4.93)	0.009	2.01 (1.01 - 4.03)	0.048
Both	8 (3.4%)	63 (1.4%)	5.66 (2.11 - 15.17)	0.001	4.21 (1.53 - 11.59)	0.005
No of antipsychotic scripts received: (within past 3 months)						
1	96 (41.4%)	1,504 (34.6%)	2.82 (1.41 - 5.65)	0.003	2.19 (1.08 - 4.42)	0.029
≥2	99 (42.7%)	1,452 (33.4%)	3.01 (1.5 - 6)	0.002	2.44 (1.21 - 4.93)	0.013
No of different antipsychotic drugs received: (within past 3 months)						
1	178 (76.7%)	2,857 (65.6%)	2.74 (1.39 - 5.4)	0.004	2.19 (1.1 - 4.36)	0.025
≥2	17 (7.3%)	99 (2.3%)	7.68 (3.32 - 17.75)	<0.001	5.87 (2.48 - 13.89)	<0.001
DDDs: (within past 3 months)						
<15	82 (35.3%)	1,440 (33.1%)	2.48 (1.23 - 5)	0.011	1.96 (0.96 - 3.99)	0.064
≥15	113 (48.7%)	1,516 (34.8%)	3.31 (1.66 - 6.6)	0.001	2.62 (1.3 - 5.25)	0.007
Formulation: (within past 3 months)						
Oral	188 (81.0%)	2,822 (64.8%)	2.95 (1.5 - 5.81)	0.002	2.34 (1.17 - 4.64)	0.016
Acute injection	5 (2.2%)	97 (2.2%)	2.28 (0.75 - 6.95)	0.147	2.05 (0.66 - 6.37)	0.215
Long-acting injection	0 (0.0%)	22 (0.5%)	-		-	

*adjusted for psychiatric hospitalization, coronary heart disease, heart failure, stroke, chronic liver disease, Parkinson’s disease, chronic renal disease, use of lithium, statins, NSAID, antiplatelet treatment, antidiabetic and antihypertensive treatment.

^∫^reference category for all odds ratios.

CI = confidence interval.

DDDs = defined daily dose.

As the vast majority of cases and controls received oral formulations, we were not able to calculate precise estimates for acute and long-acting injections.

Subgroup analyses showed that the higher risk was in women aged 65 or above (OR 4.96, 95% CI 1.55 to 15.9) (Table 3). Table 4 shows the numbers needed to treat to harm (NNH) for each category of AP exposure and the number of excess cases per 10 000 patients treated over a year. For example, the NNH for current AP exposure was 624 (404 to 1,365); for patients aged 65 year and older was 244 (178 to 388), for women was 326 (237 to 520), and for women older than 65 years was 160 (121 to 236). The corresponding numbers of excess cases of PE per 10 000 treated patients were 16 (7 to 25), 41 (26 to 56), 31 (19 to 41), and 62 (42 to 83), respectively (Table 4).

**Table 3:** **Risk of pulmonary embolism associated with antipsychotic use: subgroup analysis for age and gender**

	Adjusted* odds ratios (95% confidence interval) for current use vs past use for each subgroup		
	**age < 65**	**age ≥ 65**	**Total**
**Women**	0.77 (0.08 - 7.17)	4.96 (1.55 - 15.9)	3.94 (1.43 - 10.88)
**Men**	0.5 (0.13 - 1.93)	1.7 (0.39 - 7.31)	1.03 (0.4 - 2.68)
**Total**	0.6 (0.19 - 1.89)	3.65 (1.48 - 9.04)	2.31 (1.16 - 4.59)

*adjusted for psychiatric hospitalization, coronary heart disease, heart failure, stroke, chronic liver disease, Parkinson’s disease, chronic renal disease, utilization of lithium, statins, NSAID, antiplatlet treatment, antidiabetic and antihypertensive agents.

**Table 4:** **Numbers needed to harm and extra cases per 10 000 current users** of **antipsychotics over one year**

	**Number needed to harm (95% confidence interval)**	**Extra cases per 10 000 treated (95% confidence interval)**
**overall population**	624 (404 to 1,365)	16 (7 to 25)
**age ≥ 65**	244 (178 to 388)	41 (26 to 56)
**Women**	326 (237 to 520)	31 (19 to 42)
**age ≥ 65 and women**	160 (121 to 236)	62 (42 to 83)

### Meta-analysis of observational studies

**Figure 2:**
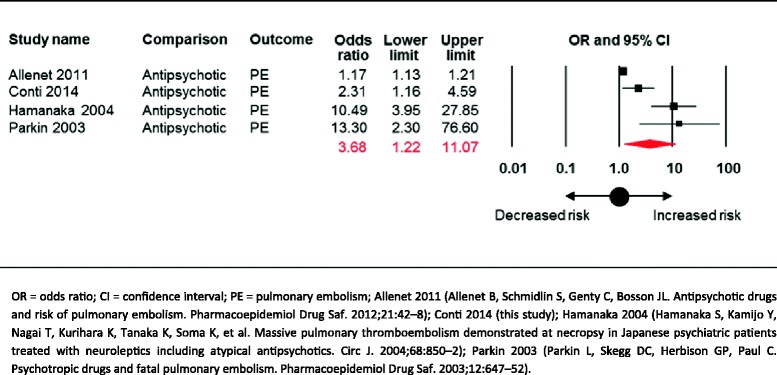
Random-effects meta-analysis on the risk of pulmonary embolism associated with exposure to antipsychotic drugs.

According to a meta-analysis published in 2013, exposure to APs did not significantly increase the risk of PE (three studies, OR 4.90, 95% CI 0.77 to 30.98), but the confidence interval was very wide and included the possibility of substantial harm. Adding the result of the present case–control study yielded a random-effects OR of 3.69 (95% CI of 1.23 to 11.07) with high-level of heterogeneity (I^2^ statistics = 90%) (Figure 2 and Additional file 3).

## Discussion

This nested case–control study, based on administrative and clinical records of individuals resident in the region of Lombardy in northern Italy, found a more than double increased risk of PE for individuals prescribed AP drugs in the past three months. The increase in risk was more than three-fold for individuals aged 65 or above, almost four times for women, and almost five time for women aged 65 or above. The overall absolute risk, however, was relatively low, with an excess of 16 extra cases of PE per 10 000 patients treated over one year; however, in women aged 65 or above the risk rises up to 62 extra cases of PE per 10 000.

Of 13 studies included in a recent systematic review and meta-analysis, only three analysed PE outcomes. The present study, therefore, significantly expands previous knowledge on this compelling issue. We showed that adding these results to those of the three observational studies [7] substantially changed the overall estimate, which now indicates that AP exposure increases of more than three times the risk of PE, with a narrower and statistically significant confidence interval.

Strengths of the study include the following. First, it was based on a large and representative population cohort, extracted from an administrative database covering a population of almost ten million inhabitants, avoiding bias from selection, non-response, or poor recall. This administrative database has been shown to have good levels of accuracy and completeness in recording prescriptions and clinical diagnoses [8,9,12,13]. In this system underreporting is unlikely, as data are used for reimbursement reasons.

Second, unlike previous studies that used composite outcome measures that include diagnostic codes related to venous thromboembolism and PE, with the advantage of increasing statistical power, we selected PE outcomes only, based on clinical grounds, as we reasoned that this is a major healthcare problem, causing over 300,000 deaths annually in Europe alone [2].

A third strength is that not only we adjusted for many potential confounding factors, but also we a priori excluded individuals with characteristics that might be associated with the outcome of interest, namely those with neoplasm, hip fractures and PE or venous thromboembolism in the year preceding the index date. We also excluded controls with any prescriptions for warfarin before the index date, as this could be treatment for a previous venous thromboembolism. According to Parker and colleagues, prescriptions for warfarin in the six weeks before diagnosis of PE did not trigger exclusion for cases, as they could plausibly be treatment for the index event itself [10]. Cases in which there was any use of warfarin earlier than this six week period, however, were excluded.

Finally, we had access to full prescription details for all AP drugs, including drug name and formulation, dose instructions, and purchase date. This enabled us to look in detail at characteristics of the drug exposure in relation to risk.

Unlike other studies, we were not able to adjust our estimates for some confounding factors such as smoking history, alcohol consumption, and body mass index, which could play a role in the development of PE outcomes [1-4]. However, in order to mitigate this limitation, we employed a nested design by incorporating a case–control approach within an established cohort of individuals who were exposed to AP drugs. This design overcomes some of the disadvantages associated with case–control studies while incorporating some of the advantages of a cohort study. In particular, confounding by indication is less likely, as all subjects were exposed to the variable of interest [14].

Similarly to other studies, we failed to adjust for some other potential confounding variables, including for example physical activity and inactivity which are well-known risk factor of PE [15]. Additionally, no adjustment was possible for medicines not reimbursed by the National health System, such as for example oral contraceptives, as these are not stored into the administrative databases. Another study limitation is that we had no possibility to access psychiatric diagnoses, which might also be associated with PE outcomes, as recently reported by Parker and colleagues [10]. We were able to include, as a confounding factor, psychiatric hospitalization, but we acknowledge that this may only be considered a proxy measure of psychiatric diagnoses.

As with all analyses of prescription databases, the lack of data on whether patients eventually took the prescribed agents should be highlighted, since a relevant proportion of the medicines prescribed for people with chronic conditions are not taken [16].

The biological mechanisms explaining the relationship between AP drugs and PE are unknown [17]. It has been suggested that all conditions associated with inactivity or immobilization may be involved. Patients might be immobilized due to the sedative properties of most AP drugs, and AP drugs may also cause weight gain which can increase inactivity and immobilization. Other biological mechanisms include raised levels of antiphospholipid antibodies and hyperhomocysteinemia, which are factors associated with PE outcomes, and might also be associated with AP exposure [17].

## Conclusions

Our findings add to the available body of evidence on the risk of PE in individuals exposed to AP drugs. The three studies available on this compelling issue are highly heterogeneous, as two found an increased risk of more than ten times, while one found a minimal increase in risk, yielding a very imprecise and statistically non significant overall estimate. Adding our study to the forest plot increased the precision of the estimate, and demonstrates that exposure to AP drugs significantly increases the risk of developing PE.

Clinical interpretation of this increased risk is difficult. The main finding of around 16 extra cases of PE per 10 000 patients prescribed AP drugs, or, in other words, the finding that a doctor would need to prescribe AP drugs to around 600 individuals to see one extra case of PE, would suggest that this risk may not be a major consideration when deciding whether AP drugs should be used. However, in specific populations, such as those aged 65 or above and women, the risk is less irrelevant, also taking into consideration that PE may be a life-threatening condition.

Additionally, very often a compelling decision is not whether AP drugs should be used but, rather, which one is less harmful. We found that both first- and second-generation AP drugs carry an increased risk of PE, but there might be differences among individual agents. We recognize that this analysis is statistically less powerful, so that for AP drugs that were not associated with an increased risk we cannot exclude a type-II error, that is failure to detect a difference, if a difference existed.

In conclusion, these data suggest that the expanding use of AP drugs to treat conditions other than approved indications should require careful consideration of the potential harmful consequences of AP drugs, including a small but significant increased risk of PE events. Patients need information on the balance of risks and benefits of these drugs before they start treatment. When treatment is needed for approved indications, clinicians should be aware that AP drugs should cautiously be used, especially among patients at high risk of PE, for example by using minimal effective doses and avoiding polypharmacy, as we observed that dose and use of two or more AP drugs substantially increased the risk. Clinicians should additionally be aware that, as with all other adverse events associated with AP drugs [18], not all drugs are equally harmful.

## Additional files

Additional file 1:
**Meta-analysis of observational studies on the risk of pulmonary embolism associated with antipsychotic exposure: methods.**
Additional file 2:
**Incidence of first hospital admission for pulmonary embolism in new users of antipsychotic drugs (n = 84,253).**
Additional file 3:
**Test for possible excessive influence of individual studies using a meta-analysis influence test that eliminated each of the included studies one at a time.**
