# Supplementary material for: Antipsychotic drug exposure and risk of pulmonary embolism: a population-based, nested case–control study
Source: BMC Psychiatry. 2015 Apr 29;15:92. doi: 10.1186/s12888-015-0479-9 (PMC4423096; doi:10.1186/s12888-015-0479-9)
Supplement: Additional file 1: — Meta-analysis of observational studies on the risk of pulmonary embolism associated with antipsychotic exposure: methods. [file 12888_2015_479_MOESM1_ESM.docx]

**Additional file 1 - Meta-analysis of observational studies on the risk of pulmonary embolism**

**associated with antipsychotic exposure: methods**

*Type of studies*

Studies were included if they were observational cohort or case-control studies that reported pulmonary embolism (PE) outcomes in individuals exposed to antipsychotic (AP) drugs (N05A group excluding lithium of the anatomical therapeutic chemical classification system) as compared with individuals unexposed or with past exposure to AP drugs. Study participants were of either sex and any age with no restrictions in terms of diagnostic categories.

*Data synthesis*

The outcome measure of this meta-analysis was the occurrence of PE in individuals exposed to AP drugs in comparison with individuals unexposed or with past exposure to AP drugs. The summary odds ratio (OR) for exposure to AP drugs was the primary measure of interest.

When possible, we pooled adjusted relative risks or OR or hazard ratios, with their 95% confidence interval, with the assumption that these are comparable measures of association given that VTE and PE are relatively rare events ^1^; otherwise we used raw data to compute unadjusted ORs ^2;3^. Visual inspection of graphs was used to investigate the possibility of statistical heterogeneity. This was supplemented using, primarily, the I-squared statistic. This provides an estimate of the percentage of variability due to heterogeneity rather than chance alone. According to Higgins and Thompson, a rough guide to interpretation is as follows: I-squared around 25% represents low heterogeneity; I-squared around 50% represents medium heterogeneity; I-squared around 75% represents high heterogeneity ^4^.

The results of studies were pooled and an overall estimate of OR was obtained from a random effects model, as this takes into account any differences between studies even if there is no statistically significant heterogeneity ^5^.

For further details on the methodology see Barbui et al. ^6^

**References**

1. Greenland S. Quantitative methods in the review of epidemiologic literature. *Epidemiol Rev* 1987;9: 1-30

2. Canonico M, Plu-Bureau, Lowe GD, et al. Hormone replacement therapy and risk of venous thromboembolism in postmenopausal women: systematic review and meta-analysis. *BMJ* 2008;336:1227-1231

3. Danesh J, Kaptoge S, Mann AG, et al. Long-term interleukin-6 levels and subsequent risk of coronary heart disease: two new prospective studies and a systematic review. *PLoS Med* 2008;5: e78

4. Higgins JP, Thompson SG. Quantifying heterogeneity in a meta-analysis. *Stat Med* 2002;21:1539-1558

5. Furukawa TA, Guyatt GH, Griffith LE. Can we individualize the 'number needed to treat'? An empirical study of summary effect measures in meta-analyses. *Int J Epidemiol* 2002;31:72-76

6. Barbui C, Conti V, Cipriani A. Antipsychotic drug exposure and risk of venous thromboembolism: a systematic review of observational studies. *Drug Safety* 2014; 37: 79-90.
