# Supplementary material for: Antipsychotic drug exposure and risk of pulmonary embolism: a population-based, nested case–control study
Source: BMC Psychiatry. 2015 Apr 29;15:92. doi: 10.1186/s12888-015-0479-9 (PMC4423096; doi:10.1186/s12888-015-0479-9)
Supplement: Additional file 2: — Incidence of first hospital admission for pulmonary embolism in new users of antipsychotic drugs (n = 84,253). [file 12888_2015_479_MOESM2_ESM.docx]

**Additional file 2 - Incidence of first hospital admission for pulmonary embolism in**

**new users of antipsychotic drugs (n = 84,253)**

|  | **Person years** | **No of events** | **Incidence / 100 000 person years** | **Rate Ratio (95% CI)** |
| --- | --- | --- | --- | --- |
| **Category of antipsychotic use:** |  |  |  |  |
| Current | 59,295 | 218 | 368 | 3.00 (1.59-5.66) |
| Recent | 20,725 | 41 | 198 | 1.62 (0.81-3.22) |
| Past^∫^ | 8,164 | 10 | 122 | 1 |
| **Total** | **88,184** | **269** | 305 |  |
| **Age ≥ 65** |  |  |  |  |
| Current | 27,087 | 186 | 687 | 4.43 (1.97-10.00) |
| Recent | 9,773 | 33 | 338 | 2.18 (0.91-5.20) |
| Past | 3,874 | 6 | 155 | 1 |
| **Total** | **40,734** | **225** | 552 |  |
| **Age < 65** |  |  |  |  |
| Current | 32,209 | 32 | 99 | 1.07 (0.38-3.01) |
| Recent | 10,951 | 8 | 73 | 0.78 (0.24-2.60) |
| Past | 4,290 | 4 | 93 | 1 |
| **Total** | **47,450** | **44** | 93 |  |
| **Female** |  |  |  |  |
| Current | 32,963 | 149 | 452 | 4.33 (1.78-10.56) |
| Recent | 12,037 | 27 | 224 | 2.15 (0.83-5.58) |
| Past | 4,793 | 5 | 104 | 1 |
| **Total** | **49,794** | **181** | 363 |  |
| **Male** |  |  |  |  |
| Current | 26,332 | 69 | 262 | 1.77 (0.71-4.38) |
| Recent | 8,687 | 14 | 161 | 1.09 (0.39-3.02) |
| Past | 3,371 | 5 | 148 | 1 |
| **Total** | **38,390** | **88** | 229 |  |
| **Age ≥ 65 and Female** |  |  |  |  |
| Current | 17,702 | 138 | 780 | 4.94 (1.83-13.35) |
| Recent | 6,410 | 25 | 390 | 2.47 (0.86-7.10) |
| Past | 2,535 | 4 | 158 | 1 |
| **Total** | **26,647** | **167** | 627 |  |
| **Age ≥ 65 and Male** |  |  |  |  |
| Current | 9,384 | 48 | 511 | 3.42 (0.83-14.09) |
| Recent | 3,363 | 8 | 238 | 1.59 (0.34-7.50) |
| Past | 1,339 | 2 | 149 | 1 |
| **Total** | **14,086** | **58** | 412 |  |
| **Age < 65 and Female** |  |  |  |  |
| Current | 15,261 | 11 | 72 | 1.63 (0.21-12.61) |
| Recent | 5,627 | 2 | 36 | 0.80 (0.07-8.85) |
| Past | 2,258 | 1 | 44 | 1 |
| **Total** | **23,147** | **14** | 60 |  |
| **Age < 65 and Male** |  |  |  |  |
| Current | 16,948 | 21 | 124 | 0.84 (0.25-2.81) |
| Recent | 5,324 | 6 | 113 | 0.76 (0.19-3.05) |
| Past | 2,031 | 3 | 148 | 1 |
| **Total** | **24,303** | **30** | 123 |  |

**Supplement 1. (continued)**

|  | **Person years** | **No of events** | **Incidence / 100 000 person years** | **Rate Ratio (95% CI)** |
| --- | --- | --- | --- | --- |
| **Formulation:** |  |  |  |  |
| Oral | 56,184 | 209 | 372 | 3.04 (1.61-5.73) |
| acute injection | 1,760 | 5 | 284 | 2.32 (0.79-6.79) |
| long-acting injection | 674 | 1 | 148 | 1.21 (0.16-9.46) |
| **Current use by substance:** |  |  |  |  |
| QUETIAPINE | 22,200 | 107 | 482 | 3.93 (2.06-7.52) |
| OLANZAPINE | 12,608 | 22 | 174 | 1.42 (0.67-3.01) |
| RISPERIDONE | 6,119 | 13 | 212 | 1.73 (0.76-3.96) |
| HALOPERIDOL | 4,888 | 40 | 818 | 6.68 (3.34-13.36) |
| ARIPIPRAZOLE | 4,015 | 5 | 125 | 1.02 (0.35-2.97) |
| AMISULPRIDE | 1,630 | 6 | 368 | 3.01 (1.09-8.27) |
| LEVOSULPIRIDE | 1,299 | 3 | 231 | 1.89 (0.52-6.85) |
| CLOZAPINE | 1,204 | 3 | 249 | 2.03 (0.56-7.39) |
| CLOTIAPINE | 957 | 11 | 1150 | 9.39 (3.99-22.10) |
| CLORPROMAZINE | 501 | 2 | 399 | 3.26 (0.71-14.87) |
| LEVOMEPROMAZINE | 494 | 1 | 203 | 1.65 (0.21-12.92) |
| PERICIAZINE | 286 | 1 | 349 | 2.85 (0.37-22.29) |
| FLUFENAZINE | 61 | 1 | 1643 | 13.4 (1.72-104.76) |
| Antipsychotic polypharmacy | 677 | 3 | 443 | 3.62 (1.00-13.14) |

^∫^reference category for all odds ratios

CI = confidence interval
