# Supplementary material for: Antipsychotic drug exposure and risk of pulmonary embolism: a population-based, nested case–control study
Source: BMC Psychiatry. 2015 Apr 29;15:92. doi: 10.1186/s12888-015-0479-9 (PMC4423096; doi:10.1186/s12888-015-0479-9)
Supplement: Additional file 3: — Test for possible excessive influence of individual studies using a meta-analysis influence test that eliminated each of the included studies one at a time. [file 12888_2015_479_MOESM3_ESM.docx]

**Additional file 3 - Test for possible excessive influence of individual studies using a meta-analysis influence test that eliminated each of the included studies one at a time**

**
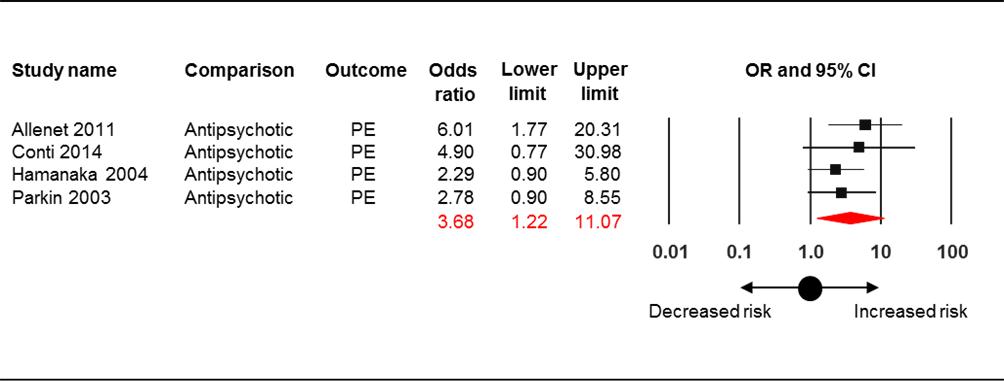
**

OR = odds ratio; CI = confidence interval; PE = pulmonary embolism; Allenet 2011 (Allenet B, Schmidlin S, Genty C, Bosson JL. Antipsychotic drugs and risk of pulmonary embolism. Pharmacoepidemiol Drug Saf. 2012;21:42–8); Conti 2014 (this study); Hamanaka 2004 (Hamanaka S, Kamijo Y, Nagai T, Kurihara K, Tanaka K, Soma K, et al. Massive pulmonary thromboembolism demonstrated at necropsy in Japanese psychiatric patients treated with neuroleptics including atypical antipsychotics. Circ J. 2004;68:850–2); Parkin 2003 (Parkin L, Skegg DC, Herbison GP, Paul C. Psychotropic drugs and fatal pulmonary embolism. Pharmacoepidemiol Drug Saf. 2003;12:647–52).
